# *In-Vitro* Approaches to Predict and Study T-Cell Mediated Hypersensitivity to Drugs

**DOI:** 10.3389/fimmu.2021.630530

**Published:** 2021-04-13

**Authors:** Sean Hammond, Paul Thomson, Xiaoli Meng, Dean Naisbitt

**Affiliations:** ^1^ MRC Centre for Drug Safety Science, Department of Molecular and Clinical Pharmacology, University of Liverpool, Liverpool, United Kingdom; ^2^ ApconiX, Alderley Park, Alderley Edge, United Kingdom

**Keywords:** drug hypersensitivity, *in-vitro*, preclinical, predictive, T-cell, immunogenicity

## Abstract

Mitigating the risk of drug hypersensitivity reactions is an important facet of a given pharmaceutical, with poor performance in this area of safety often leading to warnings, restrictions and withdrawals. In the last 50 years, efforts to diagnose, manage, and circumvent these obscure, iatrogenic diseases have resulted in the development of assays at all stages of a drugs lifespan. Indeed, this begins with intelligent lead compound selection/design to minimize the existence of deleterious chemical reactivity through exclusion of ominous structural moieties. Preclinical studies then investigate how compounds interact with biological systems, with emphasis placed on modeling immunological/toxicological liabilities. During clinical use, competent and accurate diagnoses are sought to effectively manage patients with such ailments, and pharmacovigilance datasets can be used for stratification of patient populations in order to optimise safety profiles. Herein, an overview of some of the *in-vitro* approaches to predict intrinsic immunogenicity of drugs and diagnose culprit drugs in allergic patients after exposure is detailed, with current perspectives and opportunities provided.

## Introduction

Immune-mediated idiosyncratic adverse drug reactions constitute an existential threat to prospective new chemical entities, encumbering the drug development process throughout its progression in an abstruse fashion. Since these iatrogenic reactions are enigmatic and rare, they are seldom encountered in the early stages of drug discovery, and often precipitate upon exposure to wider populations with potentially terminal consequences for both patients and drug. It is therefore astute to screen new therapeutics for the capacity to elicit such reactions, and attempt to eliminate compounds with unacceptable liability for hypersensitivity early in development. Much investment has been made to this end with several approaches developed, each with its advantages and limitations. Non-human *in-vivo* models (1, 2) possess obvious limitations in terms of translational relevance, and the fact that such equivalent models have been rendered obsolete in the field of cosmetics safety perhaps indicates a finite time for their application in drug safety studies.

**Figure 1:**
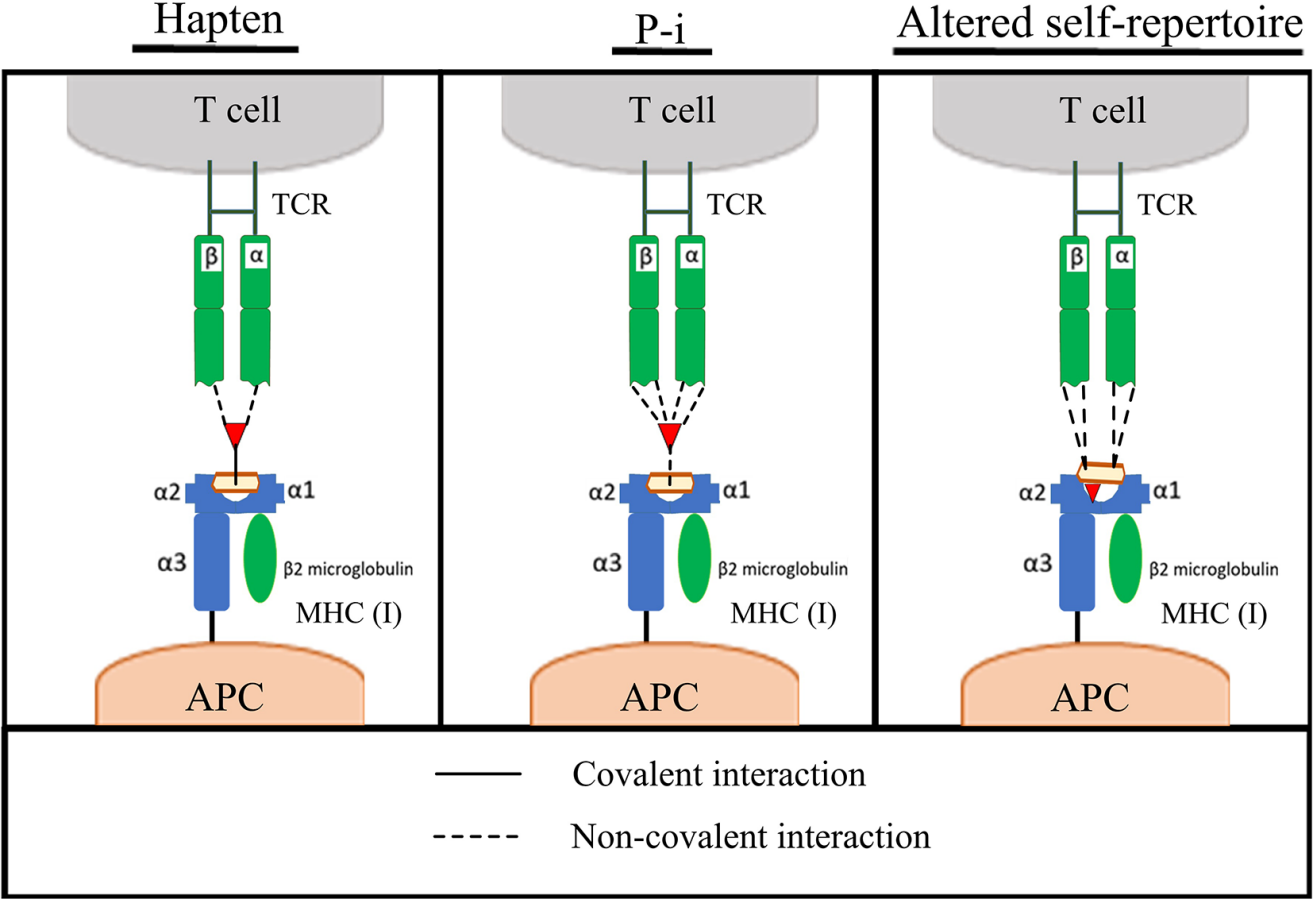
Pathways of T-cell activation by compounds. Left to right; Hapten, pharmacological interact (Pi) and altered self-repertoire hypotheses for the mechanism of antigen presentation in drug hypersensitivity. Adapted from (7, 8).

**Figure 2:**
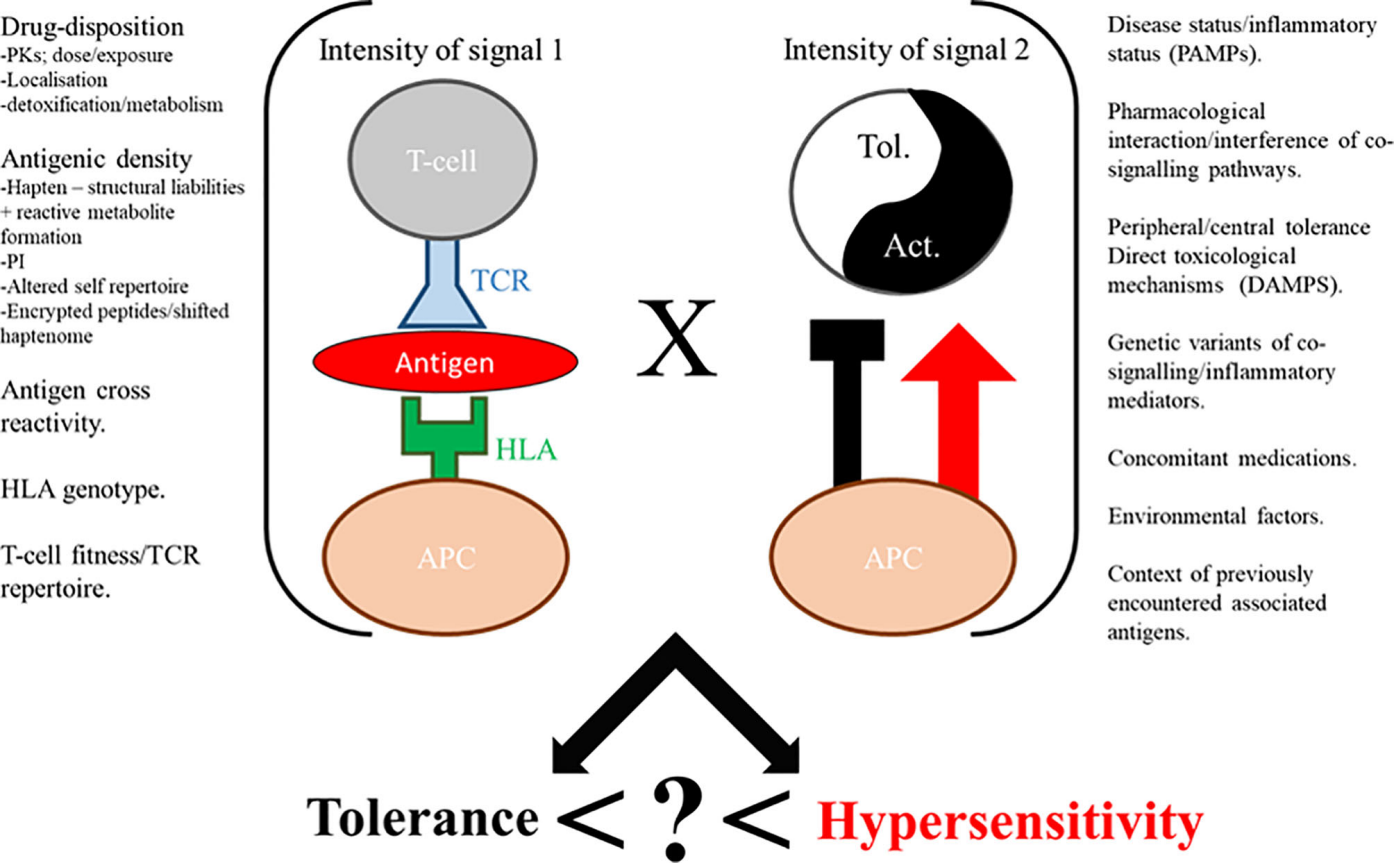
Immunological perception of drugs expressed as a function of signal 1 x signal 2 factors, with an unknown composite (?) yielding hypersensitivity. Tol. = tolerance, Act. = activation.

Despite continued efforts, understanding of hypersensitivity reactions is yet to reach satisfactory resolution. It is therefore not surprising that preclinical screening does not yet provide a blanket barrier to the progression of compounds that have the capacity to cause these reactions. The central dogma of sensitization and elicitation phases gleaned from the field of contact sensitization fundamentally holds true for drug hypersensitivity reactions (3). Indeed, there is consensus that the majority of these reactions proceed through the basic dogma of T-cell immunology; a T-cell receptor expressed on a T-cell recognizing an antigen presented in the context of human leukocyte antigen (HLA), with drug-induced perturbation of this immunological synapse and the ensuing aberrant deployment of T-cell responses a fundamental feature. Beyond this, the field dramatically diverges, with multiple pathways of antigen derivation gleaned to date; hapten (4), Pi (5), and altered self-repertoire (6), outlined in (**Figure 1**) and reviewed in detail elsewhere (7). Indeed antigen generation has been an important focus of the field for some time, and while understanding is far from complete in this area, there has been excellent progress, with some studies elegantly demonstrating how antigens can be formed in exquisite detail. Unfortunately, antigen generation is not itself the critical determinant of hypersensitivity. Rather, it appears to be a function of antigen perception and density. A simple, but helpful way to consider the induction/precipitation of such reactions is through a vaccine metaphor; broadly characterizing attributes into antigenicity (signal 1) and adjuvant potency (signal 2), with a plethora of drug and patient specific factors contributing to both (**Figure 2**). Where hypersensitivity reactions are particularly challenging is the immunological mechanisms that underpin the initiating adjuvant sequence. This aspect, embodied as the “danger hypothesis” (9, 10) is much less defined; it is heterogeneous, and probably interchangeable, but essential for an antigen perception that favours an aberrant T-cell response (11). It is known that signal 2 can be achieved via cellular stress/damage through damage associated molecular pattern (DAMP) signaling, which can be attributed to a drug by means of direct toxicological properties, or through disease/environmental factors. It can also be determined by pathogen associated molecular pattern (PAMP) signaling, as is seen with infections. Finally, what has become clear in recent times with the unpropitious outcomes seen with concomitant medication usage alongside immune checkpoint inhibitors (12–17), is that the adjuvant component is truly the dynamic and complex setting of immune regulation, and that opposing tolerance mechanisms play a critical role in determination of antigen perception. Advanced discussion on the etiology of hypersensitivity reactions is outside of the scope of this review; for extensive reading on this topic, and the mechanisms by which T-cells elicit cellular damage in the context of drug hypersensitivity the authors refer readers to a number of key reviews (7, 18–21).

These multi-mechanistic pathways of antigenicity and adjuvanticity, overlaid with the variety of tissue specific factors pertaining to localized metabolism, damage/pathogen derived signaling and cellular milieu results in extensive heterogeneity of these reactions. This heterogeneity makes preclinical assays with good coverage challenging to construct. It also translates to the challenges within the clinic in terms of clinical presentation. Indeed, manifestations are diverse, and often lack pathognomonic features (22, 23), making diagnostic certainty and effective coverage from methodologies challenging to obtain.

The imperfect classification of compounds yielded by currently available preclinical assays has resulted in the presence of many drugs with such issues within a physician’s armamentarium. Therefore, effective and safe diagnosis of hypersensitivity reactions when they do occur is paramount; in order to mitigate the re-administration of offending agents and identify liabilities of compounds in polypharmacy settings. As direct re-exposure of hypersensitive individuals is undesirable due to understandable patient anxiety and the potential for extreme risk, it is transparent that there is demand for the development of *in-vitro* methods in order to aid clinical diagnoses of hypersensitive individuals whilst mitigating re-exposure risk for suspected drugs. Hypothetically though, the ultimate goal should be the development and implementation of efficacious investigative procedures which facilitate the circumvention of hypersensitivity in early product development. Hence, this review predominantly covers the topic of *in-vitro* diagnostic assays, and provides an overview of the established and prospective efforts underway in preclinical development to circumvent the progression of compounds carrying unacceptable hypersensitivity risk profiles.

## *In-Vivo* Diagnosis/Assessment

### Drug Provocation and Skin Testing

The gold standard for diagnosis of drug hypersensitivity is the recurrence of injury upon rechallenge with the offending compound (24). Although not infallible, positive re-challenges (often following a positive de-challenge; where injury resolves upon drug cessation) provide tangible and clinically relevant evidence for or against hypersensitivity and thus whether continuation with the drug is a viable course of action. A common feature of positive re-challenge events is that the injury recrudesces in a more rapid and severe manner (25). Although beneficial for the positive identification of hypersensitivity, this phenomenon also represents a significant drawback of re-challenge; the risk of serious injury or mortality. This is highlighted by the 51% fatality rate reported for positive re-challenge events concerning the general anaesthetic halothane (26). As a result of this risk, many governing/advisory bodies issue caution when considering re-challenge where drug-induced liver injury or serious idiosyncratic adverse drug reactions are observed. A common, less hazardous approach is that of skin testing; multiple variations of skin testing exist, with the clinically utilised procedures being the skin prick test, intradermal test, patch test and photopatch test. These assays have seen clinical validation and are used routinely; for those seeking more comprehensive review of their utility, the authors refer readers to several specialist publications (27–34). Despite uptake within clinical practice, all 3 of the described skin tests possess limited sensitivity and specificity, with variable values for each parameter reported in literature (28, 35, 36). Given the limitations of skin testing, and the undesirable crux of patient exposure to a compound they are suspected to be hypersensitive to (37), there has been a longstanding necessity for minimally invasive, *in-vitro* assays that add value in diagnosis of compound hypersensitivity.

### HLA Associations and Screening

The incredible capacity for HLA genotype to predict an individual’s propensity for hypersensitivity to a selection of pharmaceuticals has brought Pharmacogenetics to the fulcrum of discussion in the field. The exquisite sensitivity of carriage of the HLA-B*57:01 “risk” allele as a determinant of an individual’s susceptibility to hypersensitivity with the nucleoside reverse transcriptase inhibitor abacavir (38–42), is the quintessential utility of this approach. Indeed, not only did the discovery of this association warrant the cost-effective and efficacious implementation of preclusive screening of prospective abacavir patients (43–46). It also laid foundations upon which mechanistic studies were able to build, eventually leading to the elucidation of a novel mechanism by which compounds can elicit hypersensitivity (6, 47). HLA screening has also been adopted for circumvention of SJS/TEN hypersensitivity reactions associated with carbamazepine, with HLA-B*15:02 featuring as the implicated allele (48). Application of pharmacogenetics is now a widespread method by which information on hypersensitivity reactions with given compounds is divulged, with a brief investigation of literature yielding no shortage of manuscripts sporting comprehensive lists of such associations. While the aforementioned HLA alleles, among others, possess exploitable odds ratios, many of the cited associations are not of consequence in terms of viable/cost effective mitigating action. The reproducibility of studies utilizing this approach has also been questionable on a number of occasions. Inversely, the fact that positive predictive values even in the most impressive of allelic associations are not 100% [abacavir exhibits only around 55% PPV (40)], alludes to the notion that confounding factors further influence susceptibility. Thus, while HLA alleles occasionally constitute a critical prerequisite, they far from guarantee the manifestation of hypersensitivity reactions (8).

Another, more fundamental issue with HLA screening in prediction of hypersensitivity is the paradoxical juxtaposition between prediction and retrospection of this approach. Crucially, a prerequisite to the delineation of risk alleles is the exposure of (often immensely proportioned) patient populations to a given pharmaceutical. Thus, patient safety is breached at the inception of these studies (an undesirable outcome in any case), therefore, HLA screening is currently confined to being a tool generated from clinical data, which on occasion has proven to powerfully contribute to the iterative process of optimization of drug safety profiles. Nevertheless, elements of this can, and have been incorporated into preclinical prospective platforms, some of which are covered below.

## *In-Vitro* Diagnostic/Investigative Assays

### LTT

**Figure 3:**
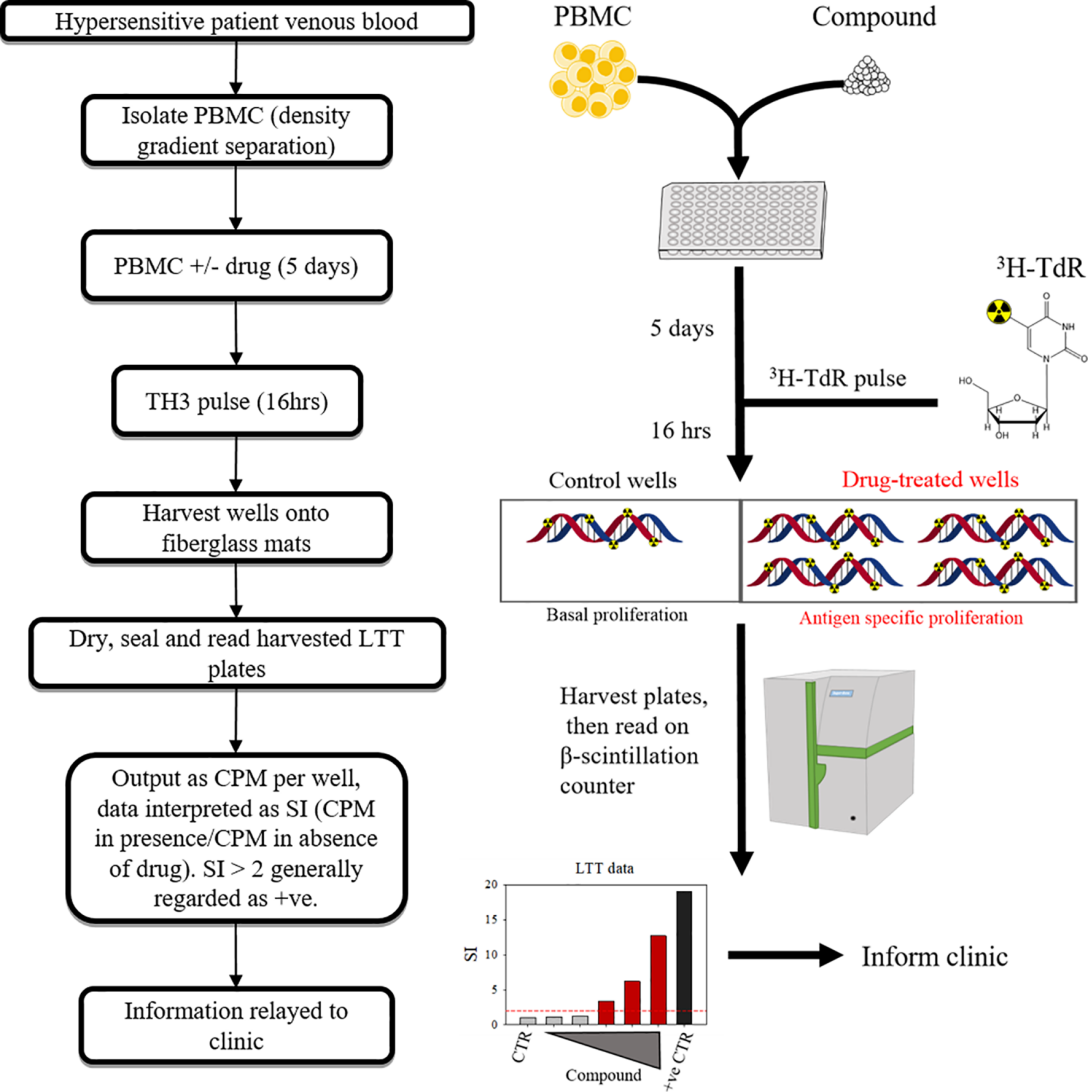
Overview of lymphocyte transformation test methodology.

The lymphocyte transformation test (LTT) has been a mainstay in the limited toolkit for *in vitro* diagnosis of hypersensititivity for around half a century, with several technical revisions since its inception (49–52). An LTT entails the culture of PBMC from an individual with suspect compounds for 6-7 days, with the output being a function of lymphoblastic transformation/proliferation. Several variations have evolved since the primitive methods described by Halpern & Amache, 1967 (53), each with benefits and drawbacks. The most prominent of these is tritiated ^3^H-thymidine incorporation (**Figure 3**). Less hazardous options include ELISA-based 5-bromo-2’-deoxyuridine incorporation method, or carboxyflourescein diacetate succinimidyl ester (CFSE) serial dilution and ki-67 expression as measured by flow cytometry. CFSE/dye dilution-type and ki-67 based assays have an additional advantage in that they can facilitate identification of the effector cell of origin (54). However both come with concerns over technical requirements, and ease of interpretation.

LTT results appear to be highly variable in terms of sensitivity and specificity, with concerns over sensitivity in particular drawing criticism which dates back to its early use (55). Confounding factors include; the clinical manifestation of the reaction (and thus the presentation-specific mechanisms), latency from reaction to test, the culprit compound in question [whether it possesses properties intrinsically inhibitory or stimulatory to T-cell proliferation e.g.; methotrexate (56)], concomitant therapy that may have bearing on the test such as immunosuppressant therapeutics, and (often overlooked) laboratory specific technique.

Clinical manifestations of drug hypersensitivity are, as discussed (8, 22, 53) heterogeneous. This heterogeneity and the delay from reaction to LTT has been posited to be absolutely critical to the validity of the test. Conventionally, a minimal interval of 3 weeks is allowed to elapse before *in vitro* tests begin, allowing elimination of both culprit drugs and immunosuppressant/anti-allergic drugs (57). Presumably this period also correlates with contraction of the adaptive response and the development of a memory component that is stabilized in terms of proliferation (as in the acute phase, highly activated PBMC may generate backgrounds which conceal responses). Further complicating this area is the contrasting time-LTT response relationship seen depending on the manifestation of the hypersensitivity reaction. Findings by Kano et al. (58), indicate that allowing time to elapse following hypersensitivity leads to opposite effects in merit of the test depending on the clinical presentation; patients with SJS/TEN exhibited prominent LTT responses in the acute phase (within 1 week), which substantially diminished upon recovery phase (>5 weeks), whilst the inverse was seen in DIHS/DRESS patients.

The transparency of compound specific variation of LTTs is epitomized by the fact that stimulation index (SI) values (and thus threshold of the definition of positive responses) are not universal. Rather, they are determined through experience with the compounds themselves, for example; while many drugs are assigned a threshold SI of >2, Beta lactams tend to be assigned a threshold of >3, and some radio contrast media responses must reach SIs >4 to be deemed positive (57). With this comes several issues which the field has failed to address universally, the first of which being inconsistent threshold SI values utilized throughout literature for compounds. For example; SIs of >2 (59), >3 (60) and >4 (57) have been adjudicated as positive responses for radio contrast media. Similarly, there remains inconsistency with Beta lactams, with SI thresholds set at >2 (61), and >3 (57). In light of this, one may be tempted to speculate that the stimulation index (and thus sensitivity and specificity of lymphocyte transformation test) is “optimized” to the data it generates. Therefore, when interpreting the sensitivity and specificity of the test cited throughout literature, this must be considered as a potential caveat. To optimize diagnosis of hypersensitivity, it would be of best interest to the field to universally agree on pre-defined SI thresholds, retrospective analyses of cumulative data from multiple laboratories may be a fruitful avenue in this respect. A second, seemingly less rectifiable issue, is that if SI threshold values may only be set retrospectively, the LTT (although useful as a diagnostic tool for hypersensitive individuals), is inherently flawed for use in determining/diagnosing potential immunogenicity of a prospective therapeutic compound in early clinical development.

Concomitant therapy is common in the aftermath of drug hypersensitivity reactions, not least due to medication taken to alleviate the reaction itself. The nature of these drugs has been suggested to influence the results of LTTs, immunosuppressant drugs such as corticosteroids (e.g.: prednisolone) have been logically suggested to inhibit proliferation and cytokine responses, patients taking > 0.2 mg/kg of such drugs are often excluded (62). Prostaglandin E2 concentration has also been posited to influence LTT results with high levels (as seen with macrophage overrepresentation in culture) and low levels (sometimes caused by use of non-steroidal anti-inflammatory drugs) reducing and enhancing LTT responses respectively (63).

Attempts to revise the LTT have included enrichment of professional antigen presenting cells within the culture, inclusion of metabolites derived from parent drug, depletion of T regulatory populations, and effector cell identification/evaluation. The enrichment of immature dendritic cells (CD14+ve) and independent pulsing with antigen prior to co-culture, demonstrated superior LTT responses in patients with amoxicillin induced maculopapular exanthema relative to standard B cells and monocytes. These modifications enhanced sensitivity, while tolerant controls remained negative- thereby maintaining specificity (64). Concordant results were also obtained from patients with heparin hypersensitivity, with the added advantage of prolongation of the sensitive detection period following the ADR (65). Antunez et al. investigated maculopapular exanthema reactions induced by iodine contrast media, finding that the CD14 enriched LTT yielded superior responses in most (but not all) patients (60), with the enrichment attenuating one patients response, and raising the baseline proliferation in at least one control. Prevailing thoughts regarding the mechanisms underpinning the superiority of dendritic cells as antigen-presenting cells (APC) in LTTs include that they are simply more adept at antigen presentation, or that as observed with nickel and DNCB (66) compounds can induce their maturation directly, in a manner that promotes immunological elicitation. The subtype of dendritic cells used to enrich the LTT must also be given consideration, as heterogeneity in response to nickel was detected when comparing Langerhans cells and circulating dendritic cells.

The role of regulatory T cells has also been scrutinized in recent times, with selective depletion of these cells from LTTs being attempted in several assays. One premise being that in some cases, although drug-specific precursors may exist, their response is suppressed by the action of regulatory T cells, hence, in their absence, the sensitivity to compounds would be unveiled. To this end, CD25+ve depletion from the culture has been utilized several times, as regulatory T cells constitutively express high levels (67, 68). In the context of allergic contact dermatitis (69), CD25 +ve depletion encouragingly yielded augmented responses to the contact allergens 2,4,6-trinitrobenzene sulfonic acid, FITC, and a-hexylcinnamaldehyde. However, responses to non-sensitizing compounds such as dimethyl sulfoxide were also augmented (albeit generally to a lesser degree). Additionally, CD25 is not exclusively highly expressed on regulatory T cells, and it is well documented that it is also expressed on B cells, activated effector T cells and certain subsets of memory T cells (68, 70–72). Therefore, the depletion of CD25 expressing cells may have unprecedented effects on the utility of the LTT (especially if PBMC are sampled in the acute phase as recommended in SJS), due to collateral removal of these other cell types, potentially resulting in false negative results through removal of drug-activated T-cells, rather than the unveiling of an otherwise suppressed response. Another modification pertaining to immune-regulation is the incorporation of immune checkpoint inhibitors. Sugita et al. (73) reported that incorporation of CTLA-4 augmented LTT sensitivity. This is an enticing prospect, but a critical question to address here would be the extent to which the enhanced sensitivity observed impacts upon the specificity of the assay. Regulatory T-cell components and regulatory pathways are likely to be critical in determining the hypersensitive status of individuals, and so false positive results would be a possible eventuality with their removal/suppression. A more detailed understanding of utility of such approaches within LTTs (perhaps even considering inter-individual components) is therefore required before it will be appropriate to routinely apply checkpoint inhibitors within such assays.

The inadequacy of the LTT in addressing metabolites derived from the parent drug may contribute to its lack of sensitivity, as some metabolites may be tissue specific. Indeed, the erratic returns from LTTs performed on drug-induced liver injury patients serve as testament to such limitations (74), as does the enhanced diagnostic performance of this assay when relevant drug metabolites are synthesized and included in LTT assessment of compounds known to undergo bio-activation (75). For metabolite coverage, several variations on LTTs have been utilized, but often these models are cumbersome; commonly relying on allogeneic metabolizing systems/cell lines such as rat/human hepatic microsomes, hepG2, hepaRG cell lines or primary hepatocytes to generate reactive metabolites, therefore restricting direct cellular contact and/or coming with the distinct caveat that allogeneic responses may undermine the assay. Nevertheless, some success has been reported with such approaches, though a degree of allogeneic response is duly reported (76, 77). In a research setting, the ultimate goal to overcome such issues would be to incorporate autologous tissues such as keratinocytes or hepatocytes which could possibly be derived through induced pluripotent stem cells, though this is impracticable for routine diagnostics. A utilitarian solution to installment of metabolizing systems within the LTT should be pursued if this assay is to realize its maximal potential in terms of diagnostic value for reactions attributable to metabolites.

Since PBMCs are the cellular input into the LTT, consideration must always be given that the output will reflect this. While use of these circulating lymphocytes is minimally invasive and is relatively practical, it does ultimately mean that translational relevance of any LTT outcome is a function of responses arising from circulating populations of lymphocytes. This equates to surveying only a small percentage of peripheral blood T-cells, which, even in totality, actually only represent around 2-2.5% of the entire T-cell complement populating an individual (78). Thus, tissue resident T-cells and specialized antigen presenting cells will be poorly (if at all) represented, and it remains imperative to consider this limitation when assessing hypersensitive statuses of patients exhibiting tissue specific responses. A similar argument may also be made for drug-associated antigens derived from tissue specific peptide repertoires. Moreover, LTTs are somewhat limited in sensitivity even if the relevant cells are present within samples. Indeed, signals from T-cells present at only low pre-cursor frequencies can be lost among backgrounds generated by heterogeneous populations within PBMCs, as has been demonstrated through T-cell cloning procedures conducted on bulk cultures from patients negative in LTTs (79–81). This is a limitation of bulk proliferation assays and indicates that there is room for improvement in terms of the threshold at which presence of T-cells actually yields detectable responses in LTTs. Finally, the quality of PBMCs available for such assays has substantial bearing on the validity of the assay; the idealistic scenario is that PBMC isolation and LTT can be performed on fresh blood within hours of phlebotomy. Frozen PBMC is reputed to be less reliable, which may be attributable to differential sensitivity to cryopreservation across cellular components (82), particularly if this significantly alters the composition of the resulting PBMC. Regardless, robust LTT responses have been observed in patient samples that have been isolated and cryopreserved, and shipped internationally to specialist laboratories. Thus, if PBMC are proficiently isolated and cryopreserved, this may represent a more pragmatic option.

Another potential avenue of consideration for the LTT is that entire formulations must be scrutinized- a recently well documented example has been that of clavulanate, which is co-formulated with amoxicillin and ticarcillin to augment antimicrobial efficacy by functioning as “cannon fodder” for bacterial beta-lactamase enzymes, acting as a substrate and thus competitively inhibiting the lactamase action on the primary active ingredient. Unfortunately however, the addition of clavulanic acid to amoxicillin precipitates drug-induced liver injury in a percentage of patients which is not eclipsed with amoxicillin alone (83). Later studies depicted distinct immunogenicity profiles for both compounds, with no cross reactivity, indicating the immunogenicity of clavulanic acid (84). Further expanding investigation of formulation leads to inclusion of excipients for a given therapeutic. Indeed, when pure substance is not available, it is recommended that injectable forms of the drug or crushed pills are used (62), albeit with the caveat of procedural artifacts. On the flipside to this however, is that batch specific immune reactions due to impurities/contamination could perhaps be identified through tablet testing, potentially absolving an active pharmaceutical ingredient of responsibility for a reaction. It is feasible that investigations of this nature could be facilitated by stability samples stored by pharma.

### Cytokine Synthesis and Secretion Detection Assays

**Figure 4:**
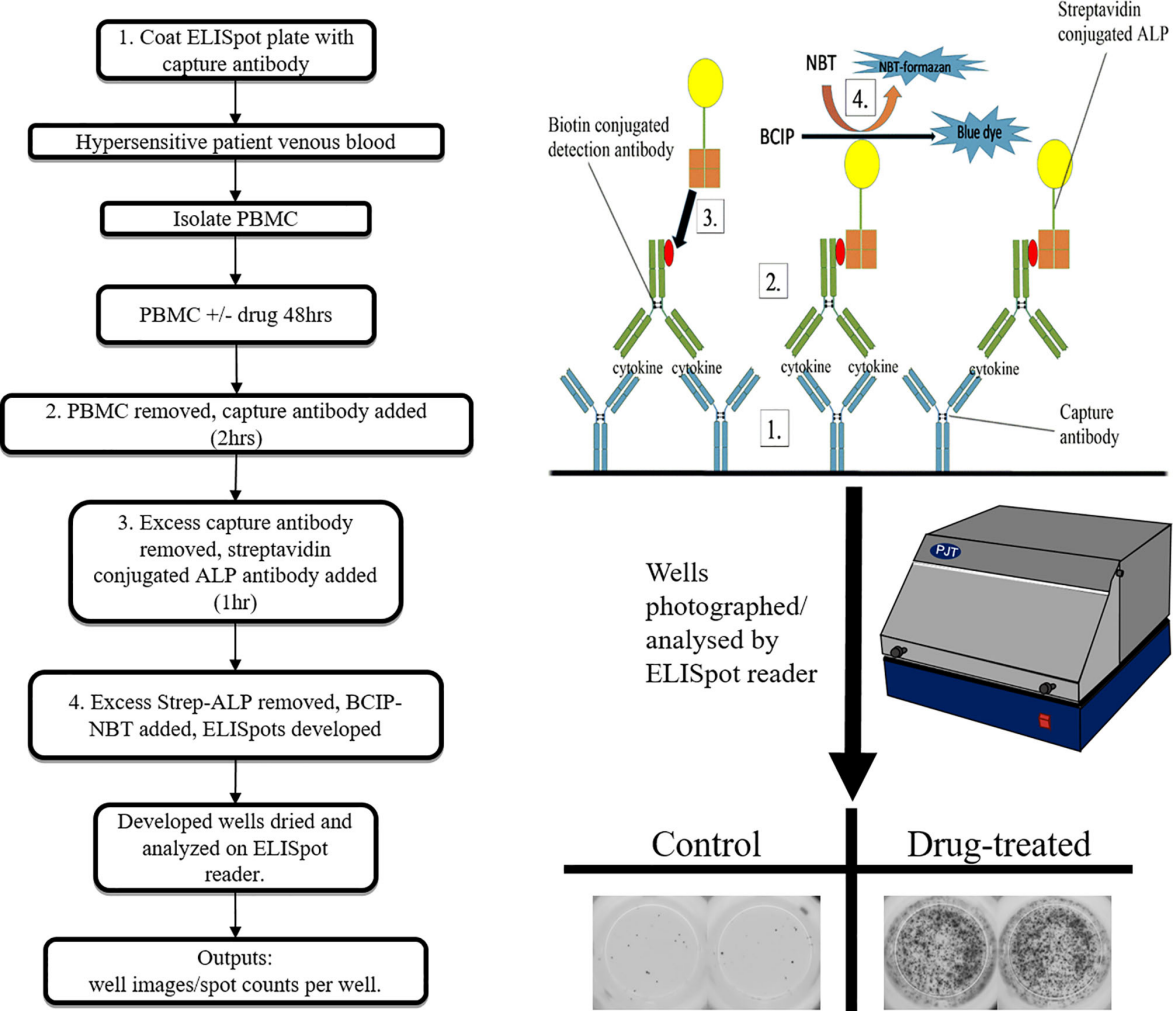
Overview of enzyme-linked immunospot (ELISpot) assay.

*In-vitro* tests targeting the function of the drug specific T-cells; cytokine/cytolytic molecule secretion assays can be both diagnostic of an individual’s hypersensitivity status, and informative in relation to pathomechanistic aspects of the reaction (85). Typical procedures used include ELISA, ELISpot (**Figure 4**), flow cytometry (intracellular cytokine staining), PCR and cytokine bead array assays. The detection parameters in such assays ultimately correspond to the synthesis and/or secretion of a given cytokine, which raises the predicament of which cytokines to use. Cytokines including IFN-γ, IL-2, IL-5, IL-13 and various cytolytic molecules such as perforin, granzyme B, and granulysin often feature, each with its merits and disadvantages depending on the clinical presentation and compound in question (57). This lead to the general recommendation that a panel of cytokines be used in order to enhance the highly variable sensitivity reported (86). Advantages of cytokine assays include the mechanistic insight provided and the relatively quick time to result (3 days), while drawbacks are the reported lack of specificity, high expense and specialist technical requirements.

### Surface Marker Expression Assays

Cluster of differentiation 69 is a member of the c-type lectin family involved in T-cell proliferation pathways (87). The upregulation of this marker (measured using flow cytometry) has therefore been utilized as an early activation marker of T-cells in delayed drug hypersensitivity and has been compared to the LTT with advantages being the quicker time to result (48 hrs rather than 1 week), the omission of the use of radioactive materials and some drug-specific peculiarities (88, 89). Markers associated with T-cell cytotoxic effector functions have also been interrogated for use in causality assessment. Intracellular granulysin expression in NKp46+ve and CD4+ve cells has been proposed in the problematic assessment of SJS/TEN (90). Similarly, surface expression of the degranulation CD107a (LAMP1) on T and NK cells, has been described for heterogeneous hypersensitivity reactions, and provides comparable mechanistic insight to that provided by ELISpot/ELISA assays (91). Other activation induced surface markers such as CD154, CD25, OX40 and PDL-1 have been used in vaccine development for some time (92, 93), notably for the detection of rare memory T-cell responses (94). Approaches such as these may therefore be of interest as sensitivity of many aforementioned assays is inadequate.

### Cytotoxicity

Inter-individual differences in the toxicological profiles of compounds (essentially detected in non-specific toxicity assays) have been linked to the hypersensitivity status of patients in several different settings. Early studies identified augmented cytotoxicity in hypersensitive patient PBMCs when co-cultured with metabolism conferring murine microsomal activating systems (95–97). Despite obvious caveats with these assays; namely the use of a xenoco-culture system, the observation of such toxicity appears to constitute a link between direct toxicity intrinsic to the individual, and the ensuing immunogenicity seen in immune-mediated idiosyncratic adverse drug reactions. Interestingly, the discrepancy in sensitivity exhibited hereditary correlation as parents of hypersensitive individuals expressed intermediate sensitivity (between controls and patients) (95), indicating a discernible role of intrinsic genetic predisposition factors. Several decades later, this approach was reinvented, employing the use of monocyte derived hepatocyte-like cells to form an autologous, metabolically competent model (98). Analogous to the results seen in the aforementioned studies, toxicity in monocyte derived hepatocyte-like cells was useful in causality assessment of idiosyncratic drug induced liver injury with comparable accuracy to that of the “gold standard” Roussel Uclaf causality assessment method (99, 100). Although this avenue of hypersensitivity investigation is in its early stages of resurrection, with other groups yet to replicate findings of these studies, it holds much promise both in utility as a diagnostic/predictive tool, and as a probe for understanding of the fundamental pre-disposing factors that influence an individual’s propensity for hypersensitivity.

### Perspective of Diagnostic Assays

To summarize, a number of diagnostic options can be pursued by a clinician in order to seek confirmation that a pharmaceutical agent should be contraindicated on the grounds of hypersensitivity. A conceptual shift has been underway for some time toward these tests being conducted *ex-vivo*, with the aim of obtaining diagnostic information while eliminating the risk of exposure for the individual in question. Unfortunately however, the battery of available *in-vitro* assays are still at various stages of development and are not yet of adequate maturity (through respective sensitivity/specificity/accessibility/standardization) to be routinely implemented into algorithms currently deployed for clinical diagnoses. It is disappointing that no functional diagnostic assay has achieved validation to date, especially given the length of time some have been studied for. A prime example of this is the LTT, which for nearly half a century has probably been the most established and clinically recognized *in-vitro* diagnostic assay, and therefore best situated for clinical validation. This is attributable to its unreliable sensitivity/specificity, and perhaps more importantly, lack of standardization. As a result, and due to the antiquated and cumbersome *in-vitro* technologies routinely used, this assay is not likely to see clinical implementation within the next decade. The widespread availability of flow cytometry probably means that any easily implementable assay will arise on this platform. Such an application also provides opportunity to multiplex features of several of the aforementioned parameters, with this type of approach likely to yield a superior, or at least more utilitarian assay. In order to facilitate this, research groups equipped to conduct these assays will need to harmonise protocols and readout thresholds in order to work collaboratively in the establishment of what would be the first legitimized *in-vitro* option for diagnosis of hypersensitivity reactions. Thereafter, efforts can be directed toward the enhancement of its sensitivity and specificity with several promising avenues discussed. As adeptly demonstrated through the peculiar retrospective/predictive properties of HLA genotyping, translational solutions are long-awaited and can be exceptionally effective in this area, but it takes standardized, translational approaches to deliver them.

As aforementioned, an idealistic goal would be to minimize or render obsolete the diagnostic field through the installation of effective preclinical screening assays. This is far from realization, with several compounds reaching clinical phases of development before programme termination in recent times, and numerous drugs in clinical circulation that have less than desirable records in terms of hypersensitivity rates. There has therefore been no shortage of incentive to gauge the intrinsic immunogenicity of prospective pharmaceuticals within preclinical development in order to select optimal lead compounds for progression. The remainder of this review therefore focuses on some of the established strategies employed within industrial settings, and outlines novel assays currently in development that may one day form part of preclinical safety studies.

## Prospective Assessment of Immunogenicity

### Structural Alerts

**Figure 5:**
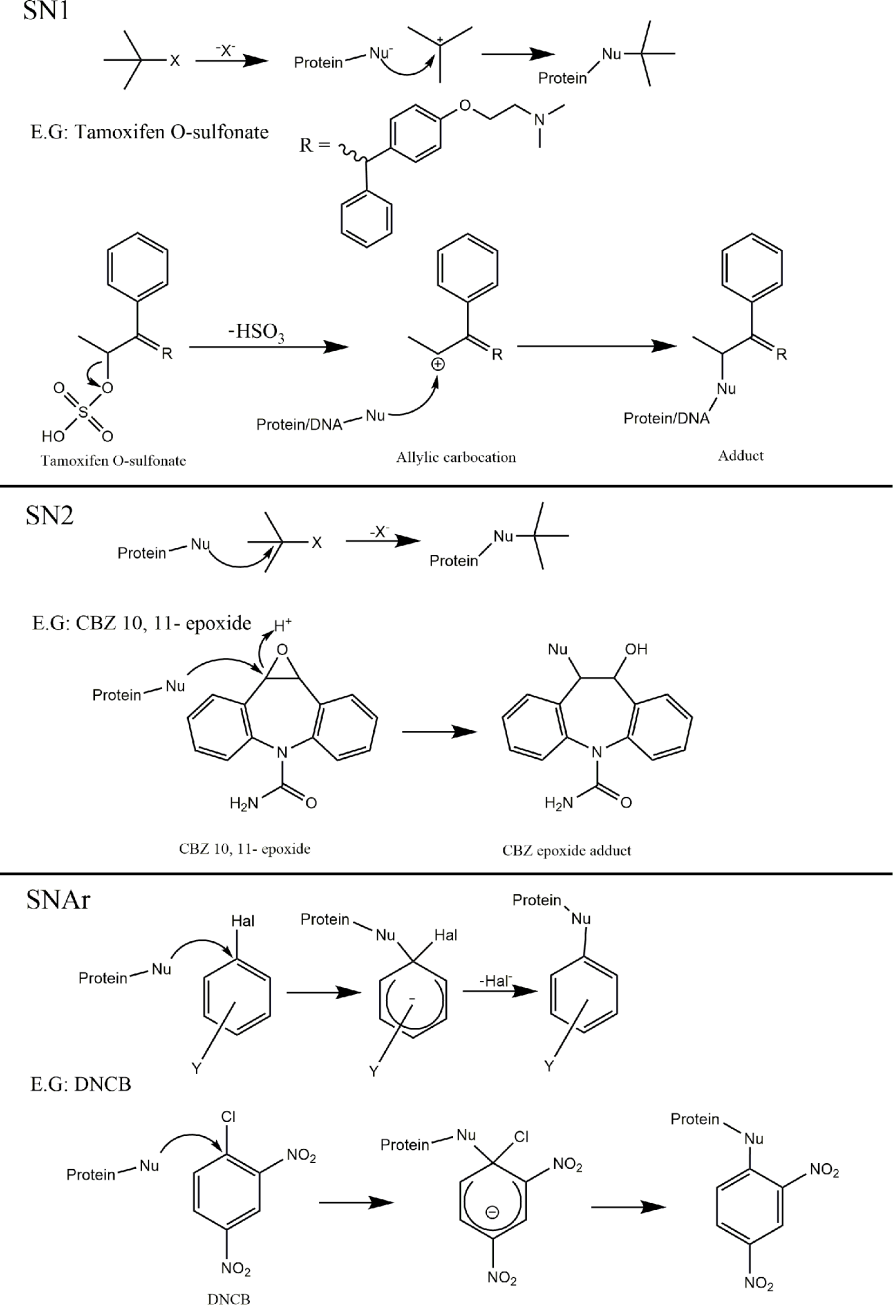
Mechanisms of covalent binding. Outline of the 6 key mechanisms by which electrophiles react with biological nucleophiles with an example compound provided for each. SN1; Tamoxifen O-sulfonate metabolite (derived from sulfonation of a-hydroxytamoxifen) can collapse yielding an allylic carbocation reactive metabolite susceptible to nucleophilic attack, resulting in protein and adducts (111–114). SN2; Carbamazepine (CBZ) 10, 11 - epoxide (reactive metabolites derived from carbamazepine) (115). SNAr; Dinitrochlorobenzene (116).

**Figure 6:**
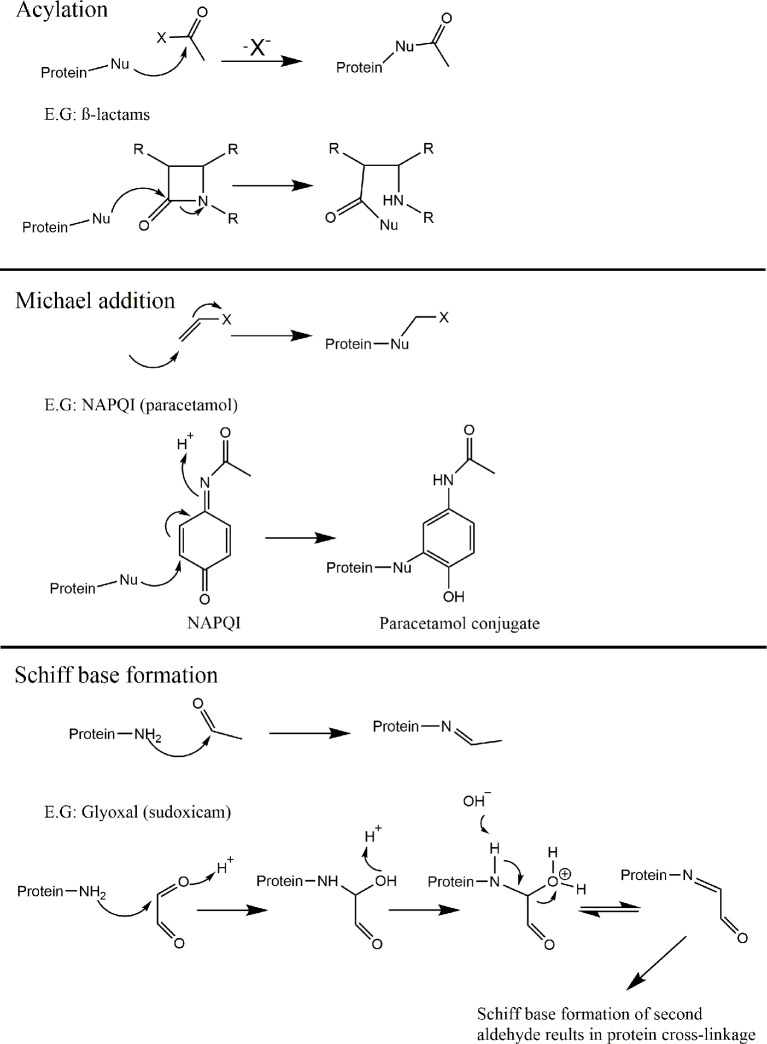
Mechanisms of covalent binding. Outline of the 6 key mechanisms by which electrophiles react with biological nucleophiles with an example compound provided for each (continued). Acylation; B-lactam containing compounds (117). Michael addition; N-acetyl-p-benzo-quinone imine (NAPQI) (reactive metabolite derived from Paracetamol) (118). Schiff base formation; glyoxal (released via bioactivation of sudoxicam) (119, 120).

Around 30 years ago, John Ashby, of imperial chemical industries, identified a codification of chemical structures that possessed genotoxic liabilities; structural alerts (101, 102). This codification was largely constructed from empirical evidence accumulated on chemical moieties responsible for covalent binding to cellular macromolecules (103–108). Since then, this basic dogma of identifying electrophilic structures that react with biological nucleophiles has undergone iterations and refinements for a variety of toxicological applications. Indeed, several *in-silico* models are now available for use as rapid, cheap, guidance tools for prediction of chemical toxicity, with the benefit of application before a compound is even synthesized (109). Understanding of the fundamental mechanisms of electrophilic reaction chemistry is therefore important in order to facilitate this philosophical shift from empirical knowledge, toward more general rules which can help inform design of such predictive tools. On this note, electrophilic reactions with biological nucleophiles have been posited to proceed through 6 basic mechanisms; S_N_1, S_N_2, S_N_Ar, acylation, Michael addition and Schiff base formation (110). For each of these, the outline of mechanism, alongside a pertinent example is provided in (**Figures 5** and **6**).

**Figure 7:**
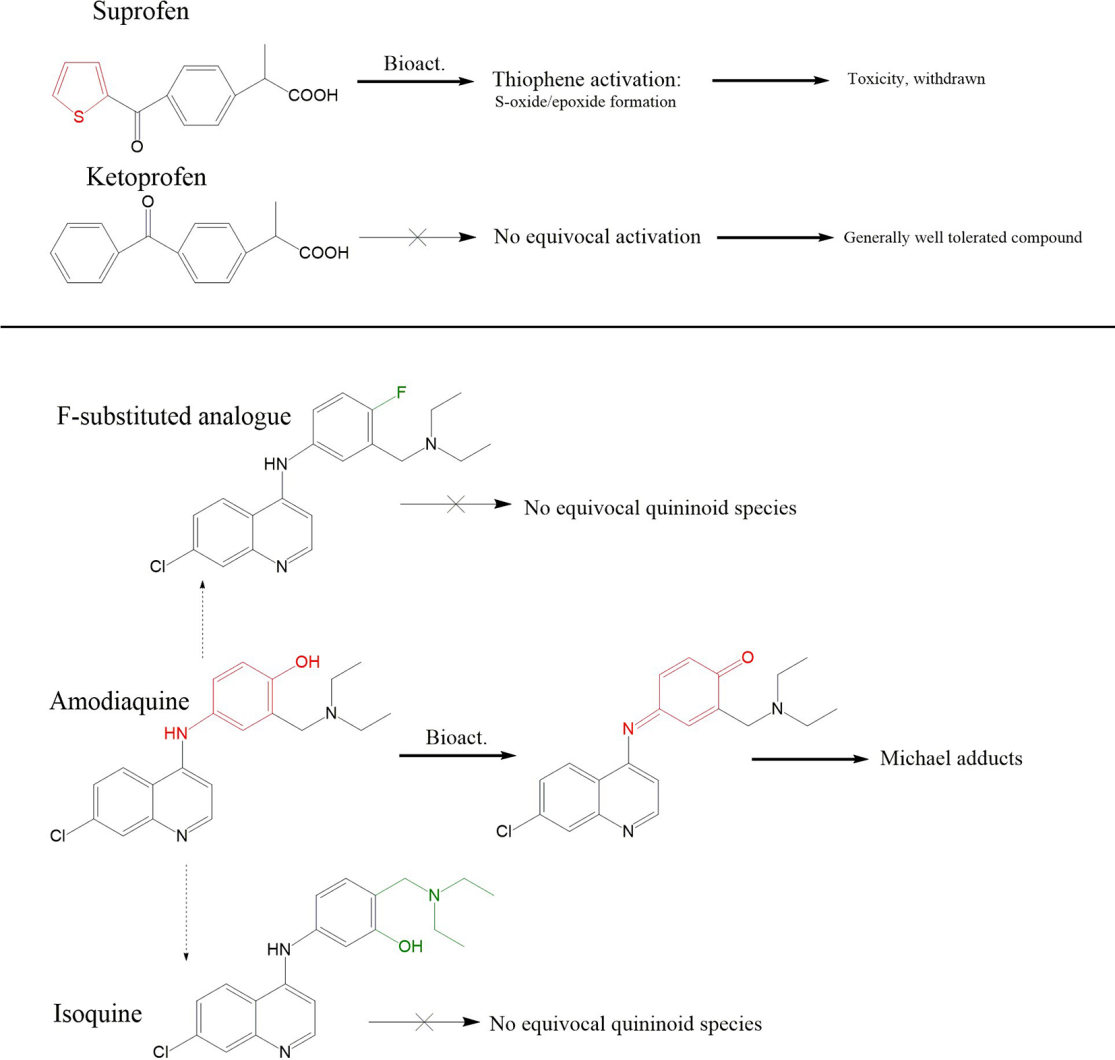
Pharmaceutical application of structural alert chemistry. Top panel; Disparity in metabolic fates of suprofen and related ketoprofen, and their downstream tolerability profiles, are generally attributed to suprofen’s possession of the thiophene ring structural alert which is capable of undergoing oxidation to S-oxides/epoxides. Ketoprofen’s phenyl ring does not undergo equivalent bio-activation. Middle panel: Iterative synthesis series of amodiaquine in pursuit of a compound with reduced ADR liabilities; Amodiaquine possesses the p-aminophenol structural alert which can be bioactivated to the Michael acceptor ACQI which is reported to be responsible for its idiosyncratic ADR liabilities in a fashion akin to paracetamol and NAPQI. Structural analogues in the form of fluorination at the 4 positions, or isomerization of the hydroxyl and diethylamino side chain leads to compounds impervious to quininoid bioactivation.

Though compounds can be intrinsically reactive as seen with β-lactam antibiotics (121), the true extent of conjugative chemistry for a given compound is often a function of its capacity to form reactive metabolites. Hence, the term toxicophore can be used interchangeably with structural alert, to refer to a compound which has reactivity imparted *via* metabolism. This has been identified as a mechanism of direct toxicity (122–124), with a direct link to hapten theory, and the propensity of compounds to cause idiosyncratic, immune-mediated reactions (125–127). The ratio of the appearance of structural alerts across drugs withdrawn/issued a black box warning relative to drugs with superior safety profiles demonstrates their unpropitious nature (128). Examples of chemical moieties that commonly feature in drugs that cause idiosyncratic toxicity include p-aminophenols or aromatic amines that can be oxidized to them (quinone reactive metabolites) (128–130), and anilines/anilides (hydroxylamine/nitroso reactive metabolites) (131–133). The logical application of such findings is therefore to design out structural alerts either in early compound design, or in an iterative fashion once the initial compound encounters idiosyncratic safety issues. One straightforward example to illustrate this approach can be found with the non-steroidal anti-inflammatory drugs suprofen and ketoprofen (**Figure 7**, top). Suprofen, which contains a thiophene structural alert, was withdrawn due to renal toxicity (134–136). Toxicological salvation can be achieved *via* replacement of the thiophene moiety present in suprofen with a phenyl ring, resulting in the safer alternative ketoprofen (137). Another example can be found in the evolution of antimalarial 4-aminoquinolones. Clinical utility of amodiaquine has been somewhat vitiated by its capacity to elicit idiosyncratic adverse drug reactions; particularly hepatotoxicity and agranulocytosis (138, 139). Amodiaquine sports an aminophenol structural alert which undergoes enzyme-mediated oxidation to form a reactive quinoneimine species which covalently binds proteins and elicits immunological responses (129, 140–145). To circumvent this deleterious bioactivation, several routes of structural redesign were pursued (**Figure 7**, bottom); including the addition of two electron accepting groups at 3′ and 5′ positions to enhance potency (146) and isomerization of the 4′-hydroxyl group with the 3′-diethylamino side chain or fluorination of the 4′-position to prevent quinonoid bioactivation (147, 148).

Structural alerts represent an anecdotal weight of knowledge through experience and should therefore be used accordingly; as a guide rather than a standard operating procedure. They far from guarantee safety; even if one was to eschew from all leads containing structural alerts, there exists several high profile examples of drugs lacking such motifs that have been withdrawn due to idiosyncratic toxicity (ximelegatran, chlormezanone, isoxicam, and pemoline) (128, 149, 150). Conversely, hit attrition concerns highlight how unsatisfactory such a parochial approach would be, with toxicophores frequently featuring in top pharmaceuticals (128, 151), and many drugs dependent on covalent mechanisms of action (128, 149, 151). Furthermore, while structural alerts indicate the possibility of a molecule covalently binding, this does not always translate; compounds containing structural alerts do not always form chemically reactive metabolites, and competing clearance pathways can trivialize the presence of alerts that do undergo bioactivation (128, 149). With regards to hypersensitivity, these examples serve to demonstrate that avoidance of structural alerts is not essential, that total body burden of chemically reactive metabolites (and therefore ensuing antigenic density) can be an important determinant, and that subtle re-design can save a lead compound.

**Figure 8:**
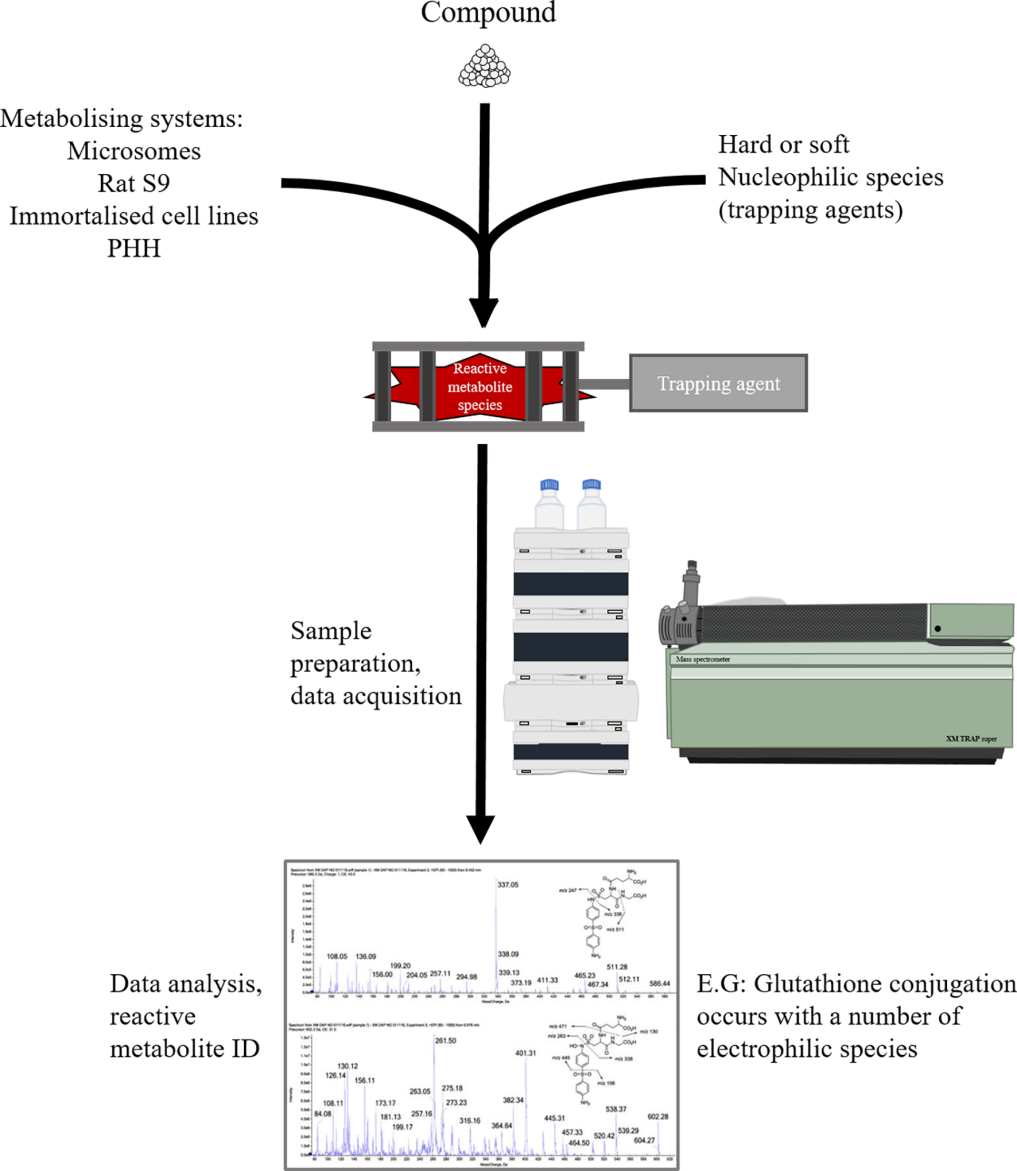
Overview of electrophilic trapping workflow.

For now, due to the emphasis on chemical reactivity with structural alerts, this type of approach currently only has utility for drugs which exert antigenicity *via* hapten/covalent binding related mechanisms. However, as patterns of drug hypersensitivity *via* the various mechanisms continue to emerge, perhaps we will eventually see inclusion of chemical codifications which confer immunogenicity, through each or all of the described antigenicity mechanisms [**Figure 1**, (8)], and/or particularly high affinity interactions for (common) constituents of the immunological synapse. One can envision that a nascent database of such “Immunocophores” could be procured from compounds that have failed at various stages of development due to idiosyncratic, immune-mediated toxicity and used to mitigate risk. Proof-of-concept iterative medicinal chemistry studies in pursuit of an analogue of abacavir devoid of hypersensitivity liabilities with preserved pharmacological action have embodied a promising prototypical approach to disconnect pharma- and immuno-cophores. Cross-disciplinary laboratories operated using *in-silico* docking models alongside functional studies to simultaneously decipher pharmacological (anti-viral) and immunological (T-cell activation) structure-activity-relationships of compound series (152, 153). Digressing from such idealistic goals, many of the following experimental assays have essentially been devised to address various aspects that lie within the void of knowledge between such conventional theoretical chemistry-based wisdom, and pragmatic transition of a compound to clinical use.

### Electrophile Trapping Assays

Reactive electrophile species formed through the bioactivation of drug candidates often exhibit insufficient stability to be directly identified through liquid chromatography-mass spectrometric methods. Hence, in order to delineate metabolites that can be derived from a given compound, a metabolically competent *in-vitro* system (cofactor fortified S9 fraction, microsomes, hepatocytes) is employed to generate reactive metabolites, which form adducts with characterised endogenous or exogenous nucleophiles, yielding “smoking gun” conjugates, providing insight into reactive metabolites formed and the mechanism by which they interact with nucleophiles (**Figure 8**). Since endogenous nucleophiles do not lend themselves to high throughput screening methods, in early compound development, small molecule nucleophilic traps are preferred. The armamentarium of these nucleophilic warheads includes the thiol-containing soft nucleophiles (glutathione, cysteine, N-acetylcysteine and 2-mercaptoethanol) for detection of soft electrophiles, and non-thiol hard nucleophiles (cyanide, semicarbazide, methoxylamine, DNA bases) for detection of hard electrophiles (154). These types of assays are mostly qualitative, but a degree of quantitative value can be added through the incorporation of radiolabelled analogues of corresponding nucleophile probes [^35^S] GSH and [^14^C] KCN (155).

Electrophile trapping assays are amenable to high throughput screening translation/automation and so feature prominently across drug discovery programmes. Despite their value and widespread utility, limitations to application of trapping in hypersensitivity prediction include: 1. the nucleophiles themselves, as any approach using exogenous nucleophiles is reliant on the assumption that these selected surrogate nucleophiles recapitulate the mechanism of adduct formation on biological macromolecules in a toxicologically/immunologically relevant fashion. 2. The physiological relevance of the somewhat simplistic *in-vitro* cultures (as detoxification pathways are not well accounted for). 3. Reactive compounds missed by such assays such as acyl glucuronides and CoA thioesters (154).

### Adductomics

Adductomics denotes a method that studies the magnitude of covalent adducts bound to tissue or blood nucleophiles which can characterise the electrophilic potential of drugs or indeed their bioactivated metabolites. This procedure involves the co-incubation of the drug-metabolite in question with conjugate proteins such as GSTP or HSA in a dose dependent manner (115). The formation of adducts can then be quantified by the use of western blotting or mass spectrometry to identify the bound amino acid residue. Protein adduction studies have been pivotal in the research of a plethora of drugs/metabolites to delineate the mechanism by which they elicit T-cell activation and whether the parent drug or a metabolite thereof exhibits the immunogenic liability (156). Jenkins et al. successfully identified the irreversible binding of flucloxacillin to HSA, in a mechanism involving nucleophilic attack of the β-lactam ring of flucloxacillin to lysine residues present on peptides (157) (**Figure 6**). This procedure has also been used for mechanistic resolution in discrimination between drugs which possess hapten functionalities and those which do not. One rather controversial example in this light is the antibiotic sulfamethoxazole, which is known to activate T-cells through a mechanism which bypasses antigen processing, namely the PI mechanism (5, 158–160) (161). However, sulfamethoxazole undergoes oxidative bioactivation to yield the metabolite nitrososulfamethoxazole. This metabolite exhibits strong reactivity toward cysteine residues, forming covalent bonds and acting as a hapten (131, 162). Accordingly, distinct patterns of T-cell activation between the relatively inert parent drug and a bioactivated metabolite can often be obtained from mechanistic studies on isolated T-cell clones (158).

Despite protein adduction of a compound not converting to a compounds liabilities in terms of capacity to elicit hypersensitivity reactions in a straightforward fashion. Drug-protein adducts have been successfully identified with antibiotics such as piperacillin (163), flucloxacillin and amoxicillin (164, 165) as well as reverse transcriptase inhibitors such as nevirapine (166). This approach has also been utilised to identify a range of peptides susceptible to covalent modification by the drug/hapten in question (121). Successful identification of such a drug-modified protein can then allow for the synthesis of designer peptides which can be integrated into T-cell assays, for analysis of their immunogenic potential (167), in a similar manner to those designed for vaccine use (168). An area of interest which may be important for the future of adductomics (with regards to both investigative and preclinical assays) will be the selected endogenous nucleophiles, and whether there may be some proteins for which covalent binding is poorly immunologically tolerated.

### Peptide Elution Studies

Within the human system HLA complexes are essential proteins which are expressed on the surfaces of many cell types which function to present peptides to T-cells. MHC class I, which presents to CD8+ T-cells, is comprised of HLA-A,B and C molecules. Meanwhile, HLA class II serves to present to CD4+ T-cells, and consists of HLA-DP, DQ and DR molecules. Many peptides from the constitutive repertoire of the host are tolerated by T-cells due to prior exposure during thymic development. However, peptides encountered thereafter such as those of viral or bacterial origin can elicit an immune response if accompanied by appropriate co-signaling and the presence of DAMPs or PAMPs (169).

**Figure 9:**
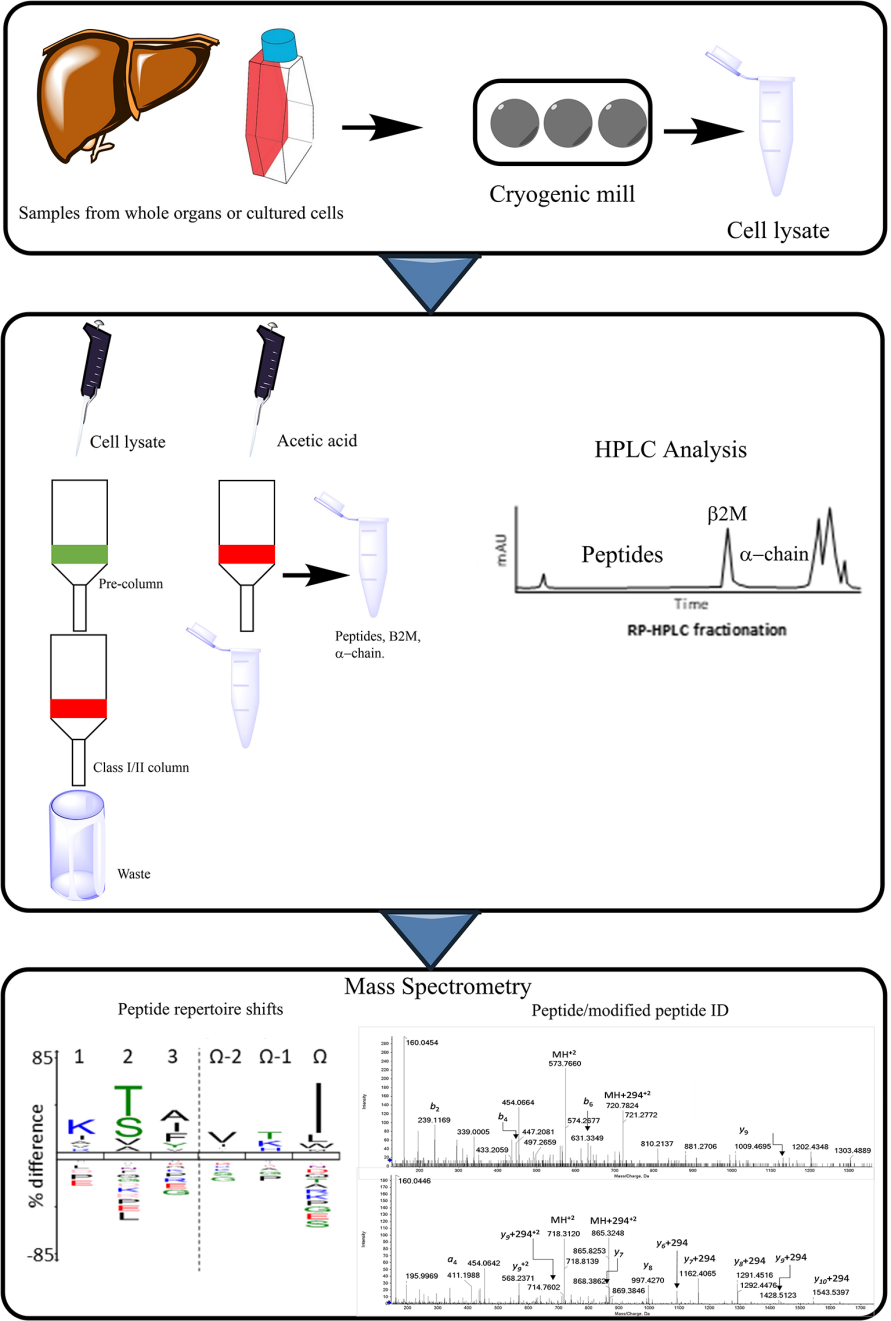
Procedural workflow of peptide elution assays. MHC complexes are purified from the samples, which can comprise of cultured cells (i.e. transfected B-cells expressing HLA allele of interest), or cells deriving from whole organs or biopsies (liver). Cell pellets can be ground using cryogenic mill and are then lysed. Immunoprecipitation takes place from the cell lysate, this occurs through running the sample through columns specific for the MHC in question, as well as a pre-column to remove non-specific binding. HPLC is then conducted to separate the MHC peptides from the larger components such as β_2_M and the alpha chain. Pooled fractions can then be analyzed *via* m/s allowing for the identification of modified peptides or an altered repertoire of peptides presented to T-cells.

Several approaches can be utilised to isolate HLA-bound peptides from a variety of cell lines. The simplest of which entails acid stripping the surfaces of cells in culture using an acidic buffer (170–172). However, this has been reported to result in high levels of contaminating peptides which can hinder the analysis of the immunopeptidome. A more commonly utilised approach involves the direct separation of solubilised HLA-complexes isolated from cell lines. This approach entails the immunoprecipitation of HLA molecules and the subsequent dissociation of the HLA-bound peptide complex which can then be analysed *via* m/s [**Figure 9**, (173, 174)].

Mass spectrometric analysis of HLA-peptide complexes has successfully identified thousands of natural MHC peptides presented on the cellular surface. These studies have been successful in the identification of the peptide binding preferences to alleles in a plethora of diseases including type 1 diabetes (175) and cancer (176, 177). Peptide elution studies have also been carried out as a pre-requisite for the study of peptide binding HLA’s, and in such cases helped to identify the N-terminal escape of 9-11 mer peptides when HLA bound (178).

It is well known that the induction of hypersensitivity reaction entails the presentation of a drug-related antigen on the surface of MHCs for scrutiny by T-cells. Indeed, this has been an area of considerable interest in recent times, including the identification of drug modified peptides or an altered repertoire of peptides on the surface of MHC. Elegant studies conducted by Illing et al. in 2012 utilised peptide elutions to positively identify a skewage toward peptides terminating in small aliphatic amino acids (I, L and V) over the conventional aromatic amino acids (F/W/P) in HLA-B*57:01+ APCs co-incubated with abacavir (179). This was achieved *via* the prolonged incubation of C1R-B*57:01 cells with abacavir followed by peptide elutions from the class I MHC and analysis by mass spectrometry (m/s). This was further reinforced in 2019 when abacavir analogues with a similar T-cell liability were found to perturb the HLA-B*57:01 peptide repertoire in a similar convention to abacavir, while those with no T-cell liability did not (180). This concept was further explored *via* the use of the β-lactam antibiotic flucloxacillin which was identified to covalently haptenate HLA-B*57:01 native peptides which were subsequently processed and presented on the MHC for T-cell recognition. This occurred through multiple mechanisms, namely, through antigen processing and direct haptenation of pre-presented peptides. Indeed, utilization of m/s analysis identified the presence HLA-B*57:01 peptides that were covalently modified with flucloxacillin haptens at lysine and arginine (181).

An obvious drawback of the peptide elution studies is the extent of technical demand; up to 1x10^9^ cells can be required for the incubation in the presence of the drug prior to conducting the elutions, mandating laborious cell culture. In the cases where specific HLA alleles are implicated, transfection of B-cell lines with the relevant alleles is standard procedure, further complicating matters, though a number of such cell lines expressing HLA alleles of interest are now commercially available. There are also procedural challenges pertaining to the translational relevance of peptides that arise through elution of transformed cell lines subject to extended culture, from which peptides are eluted in a process that may not entirely recapitulate peptides actually presented. Of considerable concern on these lines is the reported yield of peptides from such procedures (182). Further issues lie with the analysis softwares used for immunoproteomic profiling, as they exhibit shortcomings in terms of detection, particularly of drug-adducted peptides; expert mass spectrometric/adductomic analysis is therefore necessitated in many studies. Thus, the considerable technical demands, translational limitations and the level of expertise required to process analytical findings have largely confined such methods to specialist investigative studies. Peptide elution studies are therefore at the time of writing very low throughput, expensive assays which are geared toward identification of critical neoantigens (eluted peptides), and thereby the nature of culprit HLA presented T-cell epitopes associated with treatment of APCs, affording valuable insight into the mechanisms of T-cell activation by a given compound. Encouragingly, peptide elution methods feature with increasing frequency in various oncological applications such as peptide vaccination and adoptive cell transfer workflows where the field is now entering a realm of discovery in personalised/tumour personalised therapeutic approaches (183–185).

### Covalent Binding Studies

Considered as the “gold standard” and often featuring as a synergistic counterpart to trapping assays are covalent binding studies. Here, radiolabelled analogs of the candidate compound are synthesised to facilitate the measurement of covalent binding in various models. Such studies commonly feature *in-vitro* studies on human and rat liver preparations (microsomes, hepatocytes), to investigate covalent binding and interspecies translatability (154). As well as *in-vivo* models where rodent species are subject to either quantitative whole body autoradiography or radiometric analysis of harvested tissues (coupled with excretion studies) in order to determine disposition of drug-related material (186). These studies are informative in terms of qualitatively and quantitatively scrutinizing covalent binding, thereby offering insight into the extent of and localisation of hapten formation and thus which organs may be most likely targeted. However, information derived from such studies comes with several notable caveats. Firstly, custom radio-synthesis of a compound requires careful selection of radioactive atom placement to avoid metabolism induced loss, and so is an expensive pursuit, not well suited to high throughput screening. Secondly, the limitations of translational relevance of human based *in-vitro* assays as well as utility of rodents within *in-vivo* studies must always be considered. Thirdly, as with failings of electrophilic trapping and adductomics, studies of this type will not be effective in detecting compounds which confer antigenicity through non-covalent mechanisms.

Finally, there is much ambiguity as to the advisable course of action to take upon the discovery of covalent binding, with multiple confounding factors such as the lack of definitive and transparently quantifiable translation to toxicity decisions (187), with projected drug dose, purpose, and mechanism of action complicating the implementation of an isolated, binary decision. These assays are therefore to be interpreted in the context of a weight of knowledge accrued on a given chemical entity, to inform decision making in drug design, and ultimately serve to help direct drug design toward a lead optimization process that mitigates/minimalizes bio-activation.

### Enzyme Inactivation

Another avenue by which toxicity can be identified is through detection of mechanism-based inhibition of metabolic enzymes (mainly CYPs) (188). Various applications of this principle and the relevant models are described adeptly in (186). Although not proving the formation of reactive metabolites per-se, findings of enzyme inactivation are often indicative that compounds undergo bioactivation. In terms of liabilities for the culprit compound, enzyme inhibition may result from the alkylation of the enzyme (often by the reactive metabolite the enzyme catalyses the formation of), which may provoke an immunogenic response through neoantigen generation such as that seen with halothane (189) and tienilic acid (190). An important consideration with this type of assay is that it is already integrated into drug development, and may therefore shed light on potential sources of neoantigens responsible for certain tissue restricted hypersensitivity reactions (particularly idiosyncratic liver injury) and highlight the responsible enzyme for reactive metabolite formation and antigenic generation early in preclinical development.

### Antigen Presenting Cell Maturation/Activation Assays

While the antigenicity of a compound is important in terms of density/affinity/variety of antigens produced, another important component of drugs liabilities for hypersensitivity reactions may well be its capacity to generate signal 2. Indeed, classic studies have elegantly demonstrated a distinction and synergy between a chemical sensitizer and an irritant (191–193), thus, a compound’s intrinsic capacity to elicit both signal 1 and 2 contributes to its overall sensitization potential. One can consider this phenomenon in a manner akin to vaccines; while peptide epitopes are the focal point of the resulting T-cell response, co-administered adjuvants are often required to provoke immune elicitation rather than tolerance to the objective epitope.

This theme is evident within T-cell priming assays to compounds, where maturation stimuli cocktails such as LPS/TNF-α are deployed in order to mature dendritic cells prior to co-culture and facilitate T-cell priming (194, 195). From this foundation a conclusion can be drawn that a compound that possesses both qualities is less desirable than either in isolation, as such a compound is self-propagating in terms of T-cell liabilities. Certainly, assays that concentrate on a compounds capacity to promote APC maturation have proven effective in the realm of contact sensitization, with the human cell line activation test (h-CLAT) a validated and widely accepted assay routinely used for determination of sensitizer potential of prospective compounds (196–199). Intriguingly, such assays can actually distinguish between irritants and sensitizers (200). Some drugs containing structural alerts can indeed elicit direct semi-maturation of dendritic cells directly, as has been demonstrated within h-CLAT assays (196, 201) and in monocyte derived dendritic cells (64) for penicillin G and amoxicillin respectively. However, this rather appeasing correlation is afflicted with the same limitations as structural alerts, in that bioactivation can also generate chemical species capable of APC maturation as seen with nitrososulfamethoxazole (202) thus limiting application of such assays unless competent metabolizing systems are in place. Additional consideration can be given to the contiguity between danger signaling a drug may elicit through direct toxicological mechanisms, and the bearing that this may have on the interpretation of antigens and target tissue for adaptive immune sequelae. Indeed, within contact sensitization studies, response element reporters are used to detect cellular stress in assays such as keratinoSens™ (203), and combinatorial models including these types of assays are being pursued with increasing frequency (204, 205). Comparable response element/gene expression based assays have also been evaluated within hepatic models with some merit (206, 207). Investigation of the hepatic-innate immune interface for liver injury causing drugs in the form of supernatant/exosomal transfer experiments has yielded meagre returns to date with no overt increase in maturation marker expression of dendritic cells observed, although release of various cytokines was reported (208, 209), as was the basis for a communication pathway between hepatocytes and the innate immune system (210–214). These experimental platforms have paved the way for development of a new series of co-culture models that explore this interface in a fashion that may be amenable to medium throughput screening (215), offering a promising avenue for APC activation based assays to be implemented alongside conventional direct toxicological studies. Ultimately, there does appear to be potential utility for assays that focus on the intrinsic potential for a compound to generate signal 2, and they have proven useful in contact sensitizer classification. However, compounds that cause drug hypersensitivity that do act through such mechanisms appear to do so subtly, thus, current models are of inadequate sensitivity to draw robust verdicts on a compounds liability to cause hypersensitivity. In any case, for these adjuvant/perception type assays to be interpreted effectively they will likely need to be paired with one or more assays that indicate a compounds capacity to generate signal 1. It also needs to be accepted that with hypersensitivity reactions often occurring at extremely low frequencies, coincidental events that provide danger signaling; infections/trauma/co-medications/environmental factors and perception of cross-reactive antigens may play a role in at least some individuals and therefore serve to reduce or even nullify the necessity for a compound to generate an adjuvant signal in order to elicit T-cell responses.

### *In-Vitro* Priming Assays

Competent *in-vitro* assessment of the potential of small molecular weight compounds to elicit *de-novo* T-cell responses has been an aspiration within the field of hypersensitivity for some time, with establishment and validation of such screening assays currently an unmet need in drug development programmes. Early studies to this end consisted of a simple repetitive stimulation of drug-naïve donor PBMC with drug and a 48hr stimulation culture followed by a 16hr ^3^H-thymidine incorporation period conducted under IL-2 deprived conditions (216). In recent times, efforts have been made to adapt established peptide priming methods (194, 217) into a formulation which facilitates the incorporation of drug-related antigens (195). These assays, repurposed from their original application in the field of contact sensitization (218), entail the co-culture of cytokine-induced dendritic cells derived from monocytes (6 day culture, matured overnight with LPS/TNF-α) with the naïve T-cell component of PBMC in the presence of antigen for 8-14 days, followed by a re-constitution and re-challenge with a fresh batch of dendritic cells and drug antigen (195). Such procedures have been utilized in the exploration of *de-novo* priming to numerous compounds, with varying degrees of priming observed, and encouragingly, some dependency on the expression of HLA risk allele for selected drugs (219, 220). Additionally, the priming assay is sensitive to perturbation of immune-regulation, with the integration of immune checkpoint inhibitors influencing the intensity of priming to compounds (221, 222), a matter that is of increasing translational pertinence (15, 16, 18).

Unfortunately, while the *in-vitro* priming assays consistently yield robust priming responses to the paradigm compound nitrososulfamethoxazole and contact sensitizers such as bandrowski’s base, there are instances (as for the parent drug sulfamethoxazole) where they do not even appear as adept as the previously described PBMC methods at detecting drug-specific responses (216, 223). This has been attributed to a lack of sensitivity as signals from T-cells present at low precursor frequencies are lost among the “noise” generated by the bulk T-cell lines produced through T-cell priming assays, as has been demonstrated by limiting dilution and clonal characterization studies (223). Recently, this lack of sensitivity has been addressed through an additional iteration of the priming assay (224), which has resulted in experimental procedures closely aligned with those described for contact sensitization (225), which permits greater numbers of experimental replicates comprised of miniaturized priming cultures. This has facilitated detection of drug-specific responses arising from rare T-cells, albeit at the price of more turbulent baselines relative to the conventional priming assay. Immuno-regulatory aspects of the T-cell multi-well assay (TMWA) have also been evaluated, with evidence for modulation of priming to compounds by checkpoint inhibitors (224).

It is fair to consider T-cell priming assays of each format as in-development. Several limitations of these assays encumber their implementation as potential screening assays within the drug development process. The first is their sensitivity; although the TMWA represents progress in this avenue, it is still limited with many pharmaceuticals, especially compounds that do not categorise as contact sensitizers. Another limitation is that of inputting the “correct”, or rather the most immunologically relevant derivative of the drug; as with the diagnostic assays, these assays are comprised of metabolically incompetent cell types (dendritic cells and T-cells). Thus, if a metabolite’s formation is dependent on metabolically active cell types is responsible for a drug’s immunological liabilities, as is the case for many pharmaceuticals, then it is unlikely that T-cell priming assays in their current format will adequately detect immunogenicity from the input of parent drug. The detection of such responses therefore depends on; 1. The integration of a translationally relevant metabolizing system into such assays, or 2. The identification of metabolites, their synthesis and input into assays. The former of which is impeded by allogenicity/cumbersome nature of such systems, and the latter represents a challenging, expensive and possibly impractical prospect, especially regarding extensively metabolized compounds. Other limitations include the cellular input (PBMCs) as tissue resident T-cells are neglected (as with diagnostic assays), poor representation of certain T-cell responses (e.g., CD4 may predominate), and the possibility of biased effector phenotypes driven by the maturation stimuli utilized.

The weight of risk determinant that HLA allele expression contributes to T-cell responses involved in hypersensitivity is highly variable; with some drugs exhibiting extreme odds ratios to particular alleles (HLA-B*5701 and abacavir) (42) while others have no known associated HLA. An important question is whether the former represent an intractable issue when it comes to preclinical screening; the incorporation of HLA allele variants into such assays would mandate dozens of parallel assays, even to cover the most abundant alleles. Finally, the question of whether these *de-novo* responses actually do translate well to what is seen within patients is poorly defined. Within priming cultures, regulatory (amongst other) constituents of PBMC are removed and extreme inflammatory conditions are used in order to provoke T-cell responses against compounds. Indeed, the question answered from a positive assay result will almost certainly be “can” rather than “would” T-cells be activated by a given compound. Regardless, satisfactory development of T-cell priming assays would likely be a welcome addition to the barrage of available immunotoxicological assays.

### *In-Silico* Approaches

The recent emergence of nascent *in-silico* modelling systems in toxicological prediction of compounds hopefully portends a new era in the field of prediction of idiosyncratic adverse drug reactions. Systems currently available include aforementioned structural alert/chemical characteristic based softwares (109, 226–228), and models that attempt to integrate *in-vitro* findings to a toxicity assessment output (229, 230). DILIsym is perhaps the most prominent of these *in-silico* biological systems (231) and though it currently lacks an adaptive immune component, it has still exhibited utility when investigating/comparing compounds which appear to proceed through adaptive mechanisms (232–234), perhaps due to factors that propagate deployment of such abberant immunological responses. With such powerful *in-silico* methods at the disposal of the field, there are examples where modelling has been utilised even with the more complex assays such as immunopetidomics, with docking models for HLA based risk assessment of prospective compounds a particularly ambitious venture of interest (235). However, a caveat of currently employed docking studies is that they focus on only one component of the immunological synapse; the HLA, and therefore do not reflect interactions dependent on other interchangable components. Exceptionally challenging barriers exist to hinder the successful, universal, transition to prediction of signal 1 for a given compound within *in-silico* docking models through modelling of the focal point of the immunological synapse. The first is the profound polymorphism of HLA itself; to the extent that, coupled with heterozygosity of individuals, HLA genotyping can be utilized for paternal testing (236) and forensic science (237). The allelic variation is mostly restricted to residues that form the peptide binding groove, with important consequences for the respective peptide binding repertoire of each HLA (238). Second is the peptide repertoire that is expressed, which is diverse and will exhibit cell type and status specific profiles (239–241). Third is the vast heterogeneity of T-cell receptors, with clonotypic expression of TCRs shown to be important for hypersensitivity reactions occurring with a select number of drugs in the context of risk HLAs (242, 243). TCRs possess remarkable variation including that of hypervariable CDR3 region (244), and exhibit high variability in docking topology with the HLA-peptide ligand (245).

Upon successful modeling of those components, topological perturbations induced by drug and any relevant metabolites, *via* each of the known mechanisms by which small molecular weight compounds activate T-cells would need to be investigated; hapten (conjugated peptides presented), Pi (pharmacological interaction with both TCR and MHC-peptides) and altered self-repertoire (topological disruption of the HLA-peptide interaction resulting in alternative TCR specificity). Thus, while the modification of abacavir (as described in *Structural Alerts*) serves as a striking application for *in-silico* modelling, the importance of accompanying functional studies was demonstrated, and the challenges associated with redesign for circumvention of deleterious interaction with even a single HLA allele illustrates the scale of development required for these assays to come to fruition. Moreover, there is no assurance in such studies that the redesign of immunocophore implicated in HLA-B*57:01 associated hypersensitivity does not give rise to a problematic, potentially worse scenario with another HLA allele. In the future, computer systems may be developed that incorporate outputs from many of the aforementioned *in-vitro* assays to yield an estimated risk assessment based on compound performance across the board. It must be noted however, that as discussed, *in-silico* models will likely only be as good as the data provided to them. Hence, further development of existing, and inception of novel assays will likely prove imperative to optimal implementation of such systems.

### Perspective of Preclinical Assays

Great strides have been made in the last 50 years to utilise empirical evidence relating chemical structure to direct and immuno-toxicological profiles, and to use this alongside preclinical screening assays in weight of evidence decision making processes. Despite this, the process is far from perfect, and several high profile therapeutics have failed at late stages of development in recent years. The current approach in industrial drug development heavily relies on chemical properties, particularly reactivity. This has substantially contributed to better informed drug design and more effective management of the risk profiles of established compounds with hypersensitivity liabilities. However, as outlined herein, these characteristics do not directly or completely translate to biological response, and much of the focus has been on mitigating direct toxicological properties rather than immunological liabilities per-se, and so there is an unmet requirement for cell-based assays to indicate these potential risks of compounds. The unfortunate truth in the arena of preclinical assays for hypersensitivity (delayed-type hypersensitivity reactions in particular) is that there is not yet an assay with adequate predictive capacity to mitigate such risk. As such, S8 2.7 of the ICH safety guidelines offers little in terms of recommended precautionary action (246). No *in-vitro* preclinical assay exists which possesses overarching applicability across all immune-mediated hypersensitivity reactions, which perhaps reflects the heterogeneity of such reactions. A quixotic, overarching model is unachievable at this time and so a composite of existing and future assays that feed into the two signal model is likely to be key to improvement and further bridging of the gap *in-vitro.* As outlined in (**Figure 2**), there are a plethora of factors that feed into both of these attributes, while drug development workflows can focus on drug-dependent liabilities, many of these factors are drug-independent. Careful due diligence in the form of target safety assessments may shed light on potential challenges with intended populations and pharmacological effects, and help aid with construction of product-tailored models. *In-silico* methods may also be useful in this regard to incorporate population specific parameters for initiating toxicological mechanisms and also to facilitate safety margin approximations. However, it will ultimately be incredibly difficult to build idiosyncratic features into preclinical development models, and so we may need to accept there will always be unknowns in this regard with each development venture. This is where precision medicine is needed, and HLA genotyping has proven how mechanistic insight and astute pharmacovigilance can be critical (to the point of therapeutic resurrection) once a drug encounters such issues within the clinic.

## Conclusions

The prediction of hypersensitivity/idiosyncratic liabilities for a given compound in drug development and diagnosis of individuals with such ailments remain largely intractable issues. Investment required for development of new therapeutics is ascending, thus so too is the cost of attrition due to hypersensitivity. Moreover, in this era of immunological enlightenment, where pharmacological attempts to wield the immune system are becoming ever more frequent, it is becoming apparent that these therapeutics and their associated risks will bring the field to the fore of development toxicology. Better *in-vitro* assays to diagnose and predict immune liabilities of therapeutics are therefore long awaited and needed more now than ever. Half a century of investment and progress in understanding the mechanistic aspects of these reactions has yielded some great returns. As our understanding of hypersensitivity reactions continues to evolve, so too will our progression in modelling, accurate diagnosis and prediction of them in the coming decades. One anticipates that key frontiers in the immediate future will be the modernisation and harmonisation of *in-vitro* diagnostic assays, and the investment in (and composite interpretation of) biological assays that independently encapsulate antigenicity or adjuvanticity of therapeutics.

## Author Contributions

SH and PT wrote the manuscript and created the figures. XM and DN revised the manuscript. All authors contributed to the article and approved the submitted version.

## Funding

This manuscript received no direct funding.

SH and PT were funded by grants from Otsuka Pharmaceutical and Merck Pharmaceuticals respectively.

## Conflict of Interest

SH was employed by the company ApconiX.

The remaining authors declare that the research was conducted in the absence of any commercial or financial relationships that could be construed as a potential conflict of interest.
